# Hourly Origin–Destination Matrix Estimation Using Intelligent Transportation Systems Data and Deep Learning

**DOI:** 10.3390/s21217080

**Published:** 2021-10-26

**Authors:** Shahriar Afandizadeh Zargari, Amirmasoud Memarnejad, Hamid Mirzahossein

**Affiliations:** 1 School of Civil Engineering, Iran University of Science and Technology (IUST), Tehran 16846-13114, Iran; memarnejad@civileng.iust.ac.ir; 2 Department of Civil-Transportation, Imam Khomeini International University (IKIU), Qazvin 34148-96818, Iran; mirzahossein@eng.ikiu.ac.ir

**Keywords:** hourly OD demand matrix, machine learning, big data, neural network

## Abstract

Predicting the travel demand plays an indispensable role in urban transportation planning. Data collection methods for estimating the origin–destination (OD) demand matrix are being extensively shifted from traditional survey techniques to the pre-collected data from intelligent transportation systems (ITSs). This shift is partly due to the high cost of conducting traditional surveys and partly due to the diversity of scattered data produced by ITSs and the opportunity to derive extra benefits out of this big data. This study attempts to predict the OD matrix of Tehran metropolis using a set of ITS data, including the data extracted from automatic number plate recognition (ANPR) cameras, smart fare cards, loop detectors at intersections, global positioning systems (GPS) of navigation software, socio-economic and demographic characteristics as well as land-use features of zones. For this purpose, five models based on machine learning (ML) techniques are developed for training and test. In evaluating the performance of the models, the statistical methods show that the convolutional neural network (CNN) leads to the best performance in terms of accuracy in predicting the OD matrix and has the lowest error in terms of root mean square error (RMSE) and mean absolute percentage error (MAPE). Moreover, the predicted OD matrix was structurally compared with the ground truth matrix, and the CNN model also shows the highest structural similarity with the ground truth OD matrix in the presented case.

## 1. Introduction

The development propagation of cities and transportation infrastructures together with population growth have produced a wide variety of travels with different purposes at different times. Population growth and the spatial dispersal of physical developments in cities have created new centers that generate intra-city trips. The generation of new trips in new areas along with increased travels in other areas has exacerbated the problem of traffic congestion in cities. Therefore, predicting the travel demand plays a vital role in urban transportation planning.

The prepared travel behavior questionnaires and annual censuses are the cornerstones of many studies and transport models [1,2]. The traditional methods for estimating the travel demand matrix also suffer from some drawbacks. These models typically determine travel demand based on an area’s land-use and employment status [3]. Due to limited information on the conducted surveys, trip purposes are often classified into four main categories depending on whether the origin or destination of the travel is the place of home or the place of work. This simplification does not take many intra-city travels into account [3]. Previous studies have also shown that it is impossible to consider all trip purposes due to the randomness of the samples taken in the origin–destination survey [2]. In addition, survey processes are very time-consuming, costly, and labor-intensive. Thus, due to rapidly changing travel patterns of cities, conventional survey-based methods cannot be used effectively in line with these changes [4,5].

The data collected from ITSs are becoming increasingly complex and have the characteristics of big data. Large companies such as Microsoft and IBM classify the data with the three characteristics of volume, variety, and velocity as big data. Working with this big data and taking advantage of machine learning (ML) techniques provides novel solutions to solve transportation problems [6].

The methods of data collection for estimating the OD demand matrix are being shifted from traditional survey techniques (e.g., OD survey sampling) to pre-collected data stored by ITSs, e.g., data obtained from GPS, automatic number plate recognition (ANPR) cameras, smart fare card of the public transportation system, inductive loop detectors, etc. These data sources have greater temporal and spatial coverage than origin–destination survey data. Additionally, the samples collected from these data have larger dimensions [7]. In addition, these data could constantly be collected. Although the data lacks socio-economic and demographic information or even trip purposes, previous studies have highlighted the importance of using these data to improve the performance of predicting travel demand models [8]. By development in big data processing, artificial intelligence, ML, and deep learning techniques, many researchers have proposed the employment of data generated by ITSs to solve urban problems [9,10]. In particular, applying artificial neural networks in transportation studies has led to desirable results during big data analysis in travel behavior research [8,11].

It should be noted that there are several methods for comparing the predicted OD matrix with a ground truth OD matrix. These methods are mostly designed based on the statistical comparison between the value of each cell of the matrix, such as root mean square error (RMSE) [2] and mean absolute percentage error (MAPE) [12]. In addition, techniques based on the structural comparison between origin–destination matrices have also been used [13]. Besides the numerical comparison between cells, these techniques examine the travel pattern distribution between different zones in the two matrices.

This paper mainly aims to develop a model using ML techniques and the data collected from ITSs to predict trips between traffic analysis zones (TAZs) of metropolitan areas. The travel demand matrix, obtained from comprehensive urban transportation studies (CUTSs), is used as the ground truth OD matrix to test and validate the presented model. The proposed model uses big data sets collected from ITSs such as ANPR camera data, Loop detector count data at intersections, smart fare card data, GPS data of a navigation application, etc. In this study, the development of the model is conducted in the Tehran metropolis as a case study. This paper further intends to train, evaluate, and compare different ML methods for predicting the hourly OD demand matrix. In this study, different ML models are evaluated based on the prediction accuracy of test data. Furthermore, the structural similarity of the matrix estimated by ML methods based on the ground truth OD matrix is also measured. It should be mentioned that although previous studies tried to solve some parts of the OD matrix estimation problem with ML, none of them used ML methods to solve the whole problem entirely. The novelty of this paper is to consider big data sources and ML techniques to solve the whole problem of OD estimation, as well as evaluating the results by both the statistical indicators and structural comparison with the ground truth. In the following, the literature regarding the prediction of OD matrices and various techniques are addressed. The data description for developing models is then explained, and consequently, the detailed development processes and methodology are explained. Statistical and structural comparisons of the proposed models followed by conclusions are discussed in the remaining sections of this paper.

## 2. Literature Review

Estimating the OD demand matrix has always possessed a fundamental problem in transportation engineering. Although various methods have been presented to solve this problem, ML and deep learning-based methods have also been implemented to solve various transportation problems in terms of big data collected by ITSs. This section reviews the methods for estimating the OD matrix, also, ML and deep learning-based methods to solve problems related to transportation.

### 2.1. Prediction of the OD Demand Matrix

Many studies have dealt with the estimation of static OD matrices. The static OD matrix generally represents travel demand in a proposed region for a given period of time [14]. Many studies have estimated the static OD matrix from various data sources, such as traffic volumes [15,16,17,18,19], smart cards [20], cell phones [7,21], and GPS data [22,23,24]. Dynamic or time-dependent OD matrix represents travel demand in different periods. This matrix is needed for optimal intersection timing, congestion management, optimal routing, and traffic simulations [25]. The temporal correlation between the collected historical data is considered to predict dynamic OD demand matrices. In addition, the modeling methods such as the least-squares model [26] and the Kalman filter method [27] are used to estimate dynamic OD matrices. Many papers are published in which the pre-collected data are used for estimating the dynamic OD matrices and the data sources are mainly based on the aggregated and anonymous data from call detail records (CDRs) and GPS data [28,29,30].

As mentioned earlier, most of the methods use classical models based on statistical approaches to estimate the OD demand matrix. However, innovative methods based on artificial intelligence, such as artificial neural networks in deep learning, have emerged as novel alternative methods to the previous ones. These methods are discussed in the following.

### 2.2. Machine-Learning (ML) and Deep-Learning Techniques

ML techniques are usually acting as the mastermind of a system. In recent years, deep learning approaches created a significant breakthrough in various fields such as computer vision, speed recognition, and natural language processing. These methods have broken accuracy records in several areas. ML and deep learning methods have been used in various areas in the field of transportation planning, such as traffic flow prediction [31,32,33,34,35,36], traffic speed prediction [37,38,39,40], travel time prediction [41,42,43,44], and travel demand prediction [45,46]. Various ML methods, from the simplest such as support vector machine, random forest, and K-nearest neighbor to more complex ones such as multi-layer perceptron (MLP) neural network, convolutional neural network (CNN), recurrent neural network (RNN), and the extended long short-term memory (LSTM) neural network, have been employed to predict traffic flows and travel demands.

ML techniques related to travel choice models have been used for a long time (since the 1990s) [8]. Since then, various studies have been using ML methods to predict different aspects of travel (demand, mode, purpose, etc.). A comparison between the ANN model and the statistical copula-based joint model for estimating travel mode and start time showed that the neural network model is not only faster and easier to implement but also gives more accurate results [47]. Many studies have employed ML techniques to identify the mode of transportation. For example, Pirra and Diana [48] predicted tour transport mode in New York using support vector machine (SVM) from the national household travel survey, and Elhenawy and Rakha [49] combined random forest and hidden Markov model to predict transport mode using call detail record (CDR) data. The hidden Markov model and CDR data were used to estimate the activity-based travel patterns during a day [7]. As an artificial neural network, the MLP model has been used to estimate OD matrix using the pre-collected and aggregated data from Google application and has shown more favorable results than other ML models [46].

This study attempts to evaluate the potential use of different ML methods to predict hourly OD demand matrix by complementing previous studies. Using pre-collected big data from ITSs, the ability of various basic and developed ML techniques to predict a demand matrix is evaluated, and finally, the best model to predict dynamic OD demand matrix is presented.

## 3. Description of Data

The data collected for this study can be classified into six categories: (1) traffic analysis zones (TAZs) of Tehran along with spatial, demographic, socio-economic, and land-use characteristics, (2) automatic number plate recognition (ANPR) camera data, (3) Loop detector counts at intersections data, (4) smart fare cards data, (5) GPS of navigation software data, and (6) OD matrix from comprehensive urban transportation studies (CUTS) in Tehran. All of them are stored completely without duplicate data. It should be noted that the data reliability, means the data completeness and accuracy, is tested.

The characteristics of the data and the preparation process of the model are described in the following sections. It should be mentioned that the data for categories 2 to 5 were collected over a 6-month (from 23 September 2019, to 19 March 2020).

### 3.1. TAZ of Tehran with Spatial, Demographic, Socio-Economic, and Land-Use Characteristics

**Figure 1:**
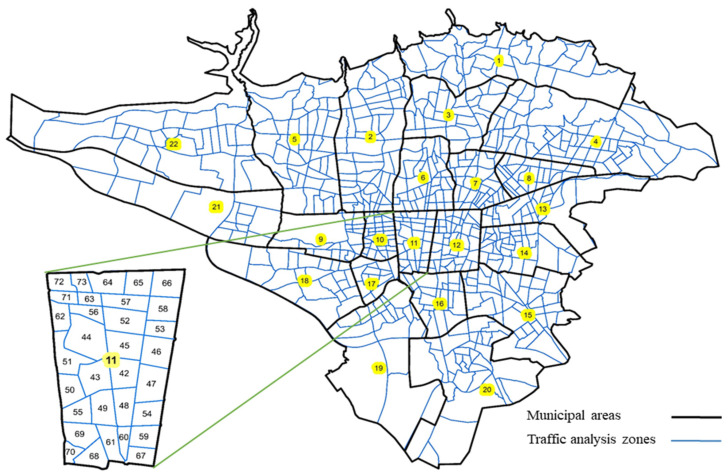
Tehran zoning map.

The metropolis of Tehran is the capital and most populous city of Iran, with a population of 8.7 million and an area of 1200 square kilometers [50]. Tehran consists of 22 municipal districts and 731 TAZs considering the surrounding areas, of which 699 TAZs are in the urban areas, and the rest are in the suburbs. The OD demand matrix of Tehran only in the city’s main area is a 699×699 matrix (Figure 1).

Studies have shown that the TAZs characteristics can estimate the number of trips that occur between OD pairs [51]. Therefore, in this study, the characteristics shown in Table 1 are considered to be the first set of input data to estimate the hourly OD demand matrix. The characteristics of traffic analysis zones in Table 1 affect the potential of a traffic zone on producing and attracting trips (trip generation). The size of the population, car ownership per capita, employee population, and commercial and administrative centers areas are trip generation factors (Variable ID 1 to 5), and distance as variable ID No. 6 affects people’s utility function in choosing the destinations. Additionally, the normalized value of each feature is considered for use in the model that presented the trip generation potential role of each feature in a trip generation. The characteristics are considered separately in the model for the origin TAZs and the destination TAZs.

### 3.2. ANPR Camera Data

**Figure 2:**
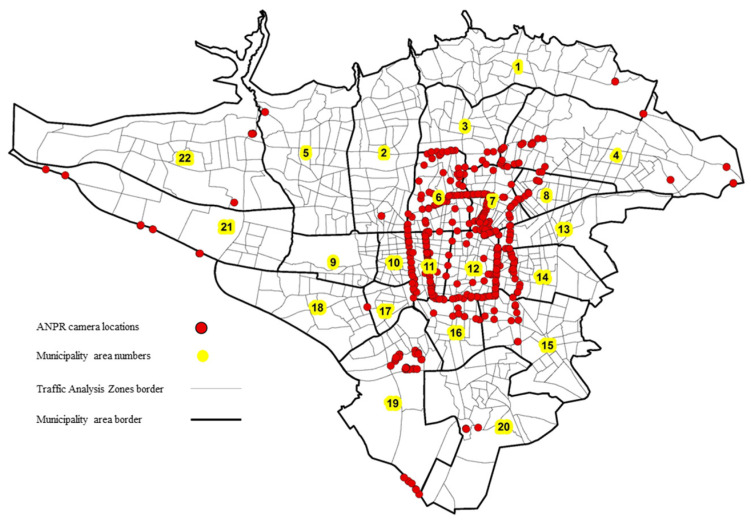
Location of ANPR cameras.

The metropolis of Tehran has been struggling with traffic congestion issues for a long time in the Central Business District (CBD) of the city. With the population growth and the increasing willingness of people to use private vehicles, this problem has been manifesting more seriously in recent years. Transportation and traffic experts in Tehran have designed and established traffic monitoring systems in the CBD of the city to apply demand management policies such as congestion pricing. Traffic demand restriction policies are applied in two layers in the CBD of Tehran. As shown in Figure 2, the entry and exit boundaries of these areas and their internal roads are equipped with ANPR cameras. The cameras capture the license plate number of all vehicles passing the camera-equipped routes. In this study, the aggregated data of these systems were used without considering the license plate number of the vehicles. The hourly data were gathered, and the hourly traffic volume at the position of each camera was assigned to the corresponding TAZ of that position. Therefore, for each TAZ, a number is calculated as the traffic count captured by ANPR cameras in each zone hourly. The data collected for six months (253,520 records consisting of 353,163,901 traffic counts) for use in the model and were averaged on weekdays and formed a 24-h data set for each TAZ.

### 3.3. Loop Detector Count at Intersections Data

**Figure 3:**
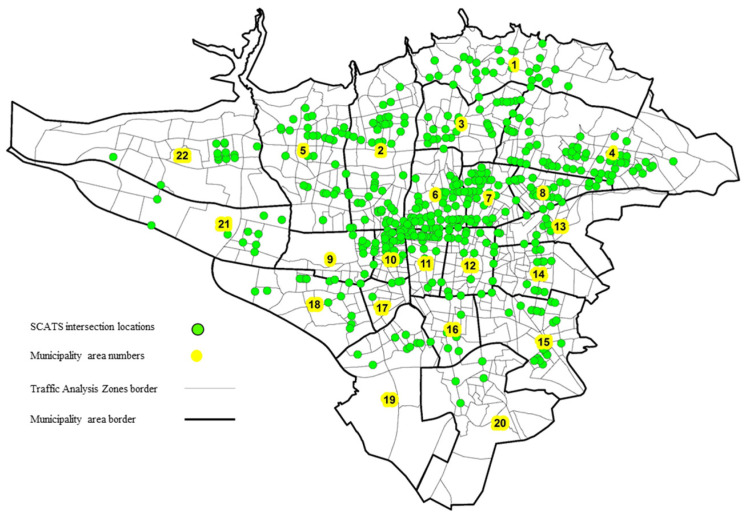
Location of adaptive real-time traffic signal control systems.

Many intersections in the metropolis of Tehran are timed by Sydney Coordinated Adaptive Traffic System (SCATS). SCATS is an adaptive real-time traffic signal control system that was first implemented by the Road and Traffic Authority (RTA) in Sydney in the early 1970s [52]. The system collects the data such as traffic volumes from each intersection, as shown in Figure 3, to apply proper timing. The data are collected by the SCATS loop detectors installed on the road surface. In this study, the traffic count data recorded at each intersection were gathered hourly and assigned to the corresponding TAZ according to the geographic locations of the intersections. The data collected for six months (1,376,068 records consisting of 1,356,175,105 traffic counts) were averaged on weekdays for use in the model and formed a 24-h data set for each TAZ. If an intersection is in the boundary of more than one zone, the traffic volumes are divided among the zones in proportion to the population.

### 3.4. Smart Fare Card Data

**Figure 4:**
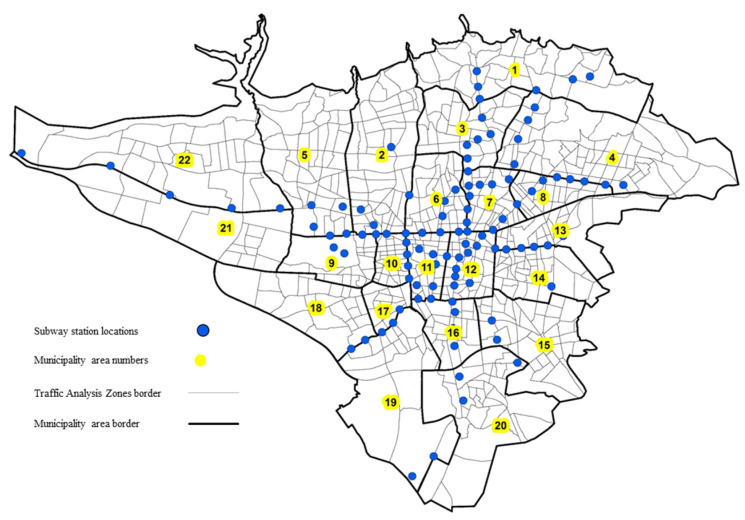
Location of subway stations.

To facilitate mobility and increase the optimal use of public transportation systems, smart cards are used in many cities for paying travel costs [53]. The metropolis of Tehran also takes advantage of this system. Therefore, smart fare cards (SFCs) provide transportation experts with useful information about the numbers of passengers in the public transport systems and the origin–destination of their travels. In this study, the number of passengers entering and exiting the subway stations was used according to the fare paid by SFCs. The hourly numbers of entries or exits from the station data were gathered anonymously from each station. The data were assigned to the corresponding TAZ according to the geographic location of subway stations, as shown in Figure 4. The data collected for six months includes 876,496 records totally which consist of 183,607,604 entries and exits. Collected data were averaged on weekdays and used in the model to form a 24-h data set for each TAZ. Hourly subway entries and exits are calculated in any TAZs. Suppose a subway station is in the boundary of more than one zone. In that case, the subway entry and exit rate is divided separately among the zones in proportion to the population.

### 3.5. GPS Navigation Software Data

Today, citizens are equipped with extensive and highly diverse portable gadgets such as smartphones, laptops, and GPS-based navigation devices that enable researchers to track their owner locations continuously. These activities can reveal travel patterns and the daily behaviors of people. Presently, researchers use these data to extract valuable information from movement patterns in cities [23]. Neshan is one of the cell phones navigation applications with more than 400 thousand active users in the metropolis of Tehran. This study used 351 million records of user location data from Neshan over six months. These data were anonymously collected while maintaining privacy policies. The recorded geographical locations were assigned to the corresponding TAZs to obtain an origin–destination matrix from the data. To estimate the matrix, some decisive rules were used to detect the termination and movement of individuals. It should be noted that numerous methods have already been used to estimate the OD matrix from mobile and GPS data [23,54,55]. The hourly matrices calculated for six months were averaged on weekdays and used in the model to form a 24-h data set for each pair of origin–destination zones. This matrix represents person trips on the network.

### 3.6. OD Matrix from Comprehensive Urban Transportation Studies (CUTS)

**Figure 5:**
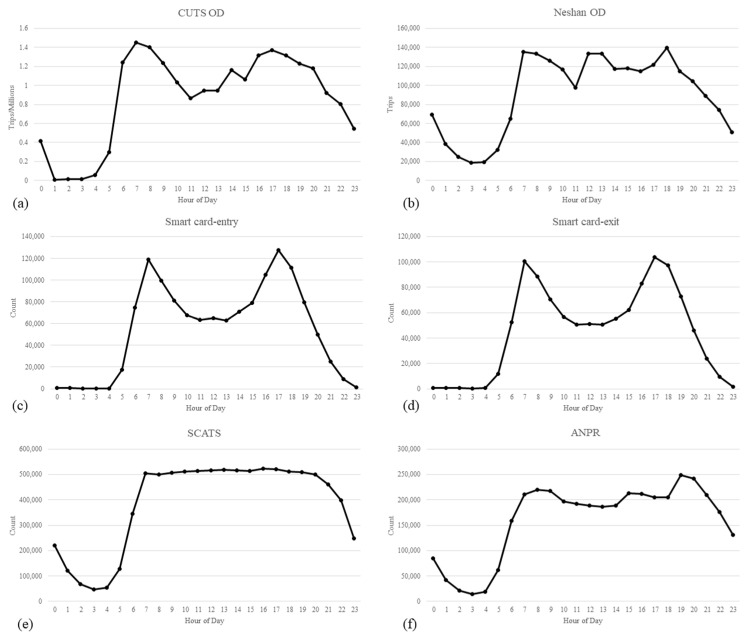
Temporal distribution of averaged data for weekdays. (**a**) temporal distribution of trips in OD matrix of CUTS, (**b**) temporal distribution of trips in OD matrix of Neshan, (**c**) temporal distribution of passengers entering to subway stations, (**d**) temporal distribution of passengers exit from subway stations, (**e**) temporal distribution of loop counts in intersections, (**f**) temporal distribution of ANPR camera count.

The comprehensive urban transportation studies (CUTS) of Tehran determined the observed OD matrix in this study. This matrix was designed on a 24-h basis and calculated based on the latest national household travel survey conducted in 2014. The OD demand matrix of citizens in the study year (2019) was calculated using activity-based models in CUTS and used as ground truth in this study. This matrix represents person trips on the network. Figure 5 shows a comparison of the temporal distribution of the collected data for all TAZs.

As shown in Figure 5, the temporal distribution of data extracted from the OD matrix of Neshan (Figure 5b), especially in the morning and evening peak hours, shows interesting similarity to the CUTS matrix (Figure 5a) which is the observed OD matrix in this study. However, a different travel pattern distribution between the two matrices can be observed in off-peak hours. Diagrams (c) and (d) show that the temporal distribution of entry to and exit from subway stations follows a similar pattern. According to diagram (a) in Figure 5, the temporal distribution of passengers entering and exiting from subway stations has a good correspondence with the number of travels at different times of a day. The peak and off-peak hours of entry to and exit from the subway are visible in the diagrams. As shown from the diagram (e) in Figure 5, the flow pattern in Tehran intersections is not remarkably similar to the previous diagrams. It seems that typically, the city’s intersections reach their performance capacity after 7 A.M. and remain at the same service level until 8 P.M. According to diagram (f) in Figure 5, the temporal distribution of traffic flow in all TAZs can be observed based on the ANPR data. Although the distribution shows the evening and morning peak hours, these times, especially in the evening, do not match the CUTS diagram. This may be due to demand management policies applied to the CBD of the city where most ANPR cameras are installed. According to these diagrams and the lack of linear correspondence between all input data and the CUTS matrix, it seems impossible to predict the OD matrix with simple ML models.

### 3.7. Selection of Input Data

The development of an efficient model significantly depends on the selection of parameters that affect the model output. Choosing the best combination of input data can significantly influence the accuracy of the final estimation of the model. Thus, the ANalysis Of VAriance (ANOVA) method is considered for finding the proper model independent variables [56]. The dependent variable is the number of trips between each TAZ from CUTS of Tehran, and independent variables, according to Table 2 are consist of; trips between TAZs in the Neshan OD matrix, time of day, the distance between TAZ zones for each pair of zones, and other dependent variables for the origin and destination TAZ zone separately including; population size, car ownership per capita, employee population, area of administrative land-use, area of commercial land-use, ANPR camera counts, SCATS loop detector counts, number of passengers entering the stations, and number of passengers exiting the stations.

In this ANOVA test, due to the large volume of input data and the inability of the personal computers (PCs) to analyze the multiple interactions between the data, only the direct relations between the input data were considered, and the 2-way and 3-way interactions were ignored. When the adjusted $R^{2}$ value for each input parameter is greater than zero (e.g., equal to b); this indicates that the parameter contributes to the description of the independent variable by the value of *b*. Therefore, when the adjusted $R^{2}$ is zero for a parameter; this parameter neither affects the description of the independent parameter in the linear form nor the nonlinear form. The effect of the model’s input variables is evaluated for origin and destination zones separately, except Neshan OD matrix, time of day, and the distance parameters, which are evaluated for a pair of zones. The values of adjusted $R^{2}$ for each of the input variables can be seen in Table 2.

**Figure 6:**
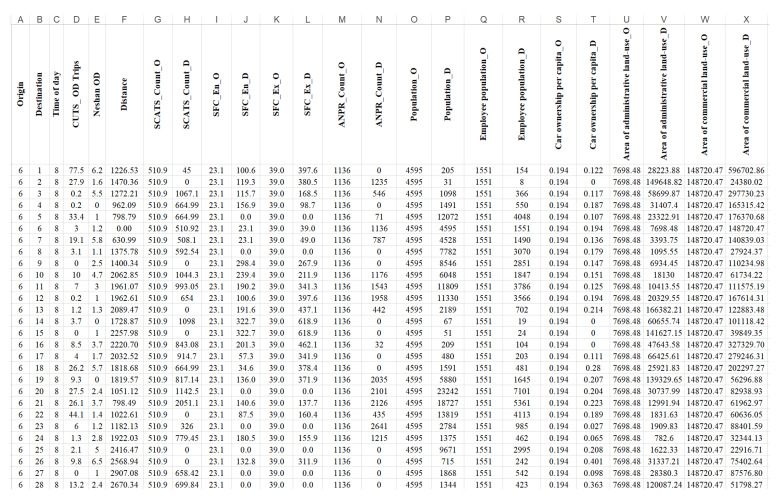
A sample snapshot of source data.

According to Table 2, all input parameters have the adjusted $R^{2}$ value greater than 0, and thus, all variables are effective in predicting the dependent variable. The effect of the variables such as the Neshan OD matrix (adjusted $R^{2}$ equal to 0.54) and the distance of zones (adjusted $R^{2}$ equal to 0.41) are significant in estimating the OD matrix. The least effect belongs to the area of administrative land-use in the origin zones (adjusted $R^{2}$ equal to 0.094). The variables collected from ITSs are generally evaluated to be more important than zones characteristics according to their higher adjusted $R^{2}$ values. For more clarification, Figure 6 illustrates a sample snapshot of input data. It represents the prepared data for trips from origin number six to other destinations at 8 a.m. All the input variables shown in Figure 6 are based on Table 2 variable name definition.

## 4. Methodology

ML techniques are basically divided into two categories: supervised methods and unsupervised methods. In supervised learning, the data intended to be predicted is also included in the model’s input data. However, there is no prior estimation of the model output in the unsupervised method, and the model is expected to determine outputs itself. As we have the ground truth, we have trained our model based on it, so, this study focused on supervised ML methods. The steps of this study are shown in Figure 6.

**Figure 7:**
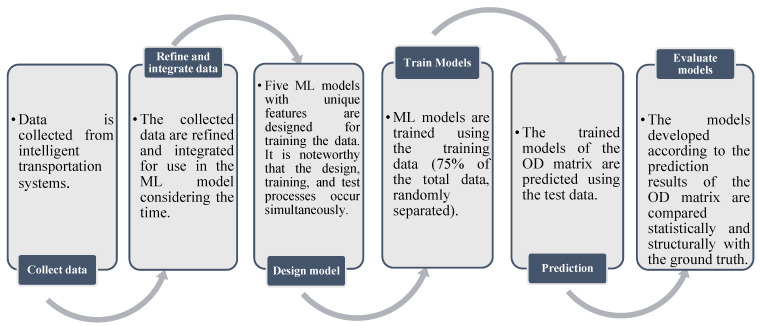
Steps of study.

According to Figure 7, all raw selected input data should be gathered from intelligent transportation systems data warehouse in the first step. These are saved completely and continuously in separate data sources. Secondly, all collected data have been prepared for entering ML models. According to Figure 6, this process contains the preparation of input data in the form of 24-h data set for each pair of origin–destination zones. The values of input data are normalized to use in the models. In the third and fourth steps, regarding the general approach for supervised ML models, the data must be divided into two categories of training data and test data (usually 25% for test and the remaining 75% for training data) [46]. The ML algorithm tries to learn the data structure by fitting the input (independent variables) and output (dependent variable) data using the set of training data. This process can be performed by minimizing the squared error between the predicted and observed output data values. In the next step, the trained model is used to predict the value of the output variables with the test data. There is always a risk of overfitting with these models, which should be minimized. Moreover, each ML model should be trained for 100 replication to choose the best models [11]. Finally, in the last step, the results of developed models, estimated OD matrices, are evaluated by statistical indicators and structural comparison with the ground truth OD matrix. Five different models of ML are described next. It should be noted that the models are summarized in this section, and further details can be found in previous studies [11,31,36,38,42,46,49,57,58,59].

### 4.1. K-Nearest Neighbor Regression

K-nearest neighbor (KNN) regression is one of the simplest ML methods. The prediction based on the observed value of KNN is performed using the distance parameter (Euclidean distance, Manhattan distance, etc.) in the multi-dimensional space of the variables. This value can be mean, median, maximum or any other statistical parameter obtained from KNN. In this study, the Scikit-Learn library in Python was used [60]. In this way, five nearest neighbors effectively predict the target values using Euclidean distance and weighted average values in the trial-and-error process.

### 4.2. Random Forest Regression

The random forest (RF) regression method was designed based on a set of random decision trees [49]. A decision tree recognizes a set of rules in the input data that can predict the target value. In this study, a decision tree relates the amount of output data to a range of input data, e.g., the relation between the range of traffic flow in TAZs and the number of trips between OD pairs. Since a decision tree usually causes overfitting of the model, a set of decision trees were trained with the random part of the data to form a random forest of decision trees. The final predicted value is the average of all predictions made by the decision trees in the RF. The Scikit-Learn library in Python was used for training and using this technique [60].

### 4.3. LightGBM (Gradient Boosting Machine) Algorithm

This framework is a fast and high-performance gradient boosting mechanism based on decision tree algorithms. It is used for ranking, classification, and many other tasks in ML. This efficient algorithm was designed for distributed applications, with the following advantages: high training speed, more efficiency, less memory usage, better accuracy, and large-scale data management capability [58]. In this study, the learning rate is considered 0.06. In addition, the early stopping round was used to avoid overfitting. In this case, by choosing a threshold value, training is stopped after several epochs without progress. LightGBM library in Python was used for training and using this technique [58].

### 4.4. Multi-Layer Perceptron (MLP) Neural Network

There are various approaches based on artificial neural network algorithms, and the MLP is one of the most common ones. This method matches the input variable layers to target variable layers. In theory, there can be an infinite number of hidden layers between the two layers. Each hidden layer contains a certain number of nodes or neurons that the user should determine. The artificial neural network allows training a highly nonlinear model; however, the initial selection of weights in the layers puts the models at risk of becoming stuck in local minima. Additional details on these models can be found in previous studies [11,31,36,42]. A fully connected architecture was designed in this study with three hidden layers, where the number of neurons in each hidden layer is 500, 500, and 200, respectively. The activation function is rectified linear unit (RELU) in the input and hidden layers, and linear in the output layers. For training and using the neural network model, the Keras library in the Python programming language was used [61].

### 4.5. Convolutional Neural Network

The convolutional neural network (CNN) is a deep learning algorithm that receives an input image and assigns importance (learnable weights and biases) to each of the objects/aspects in the image and can distinguish them from each other. The CNN algorithm requires less pre-processing than other classification algorithms. Although the filters of basic methods are performed manually, CNN acquires the ability to learn filters/features after enough training. CNN can successfully capture temporal and spatial dependencies to an image with appropriate filters. Moreover, it performs better filter architecture on image data set due to the reduced number of parameters involved and reuse of weights. In other words, the network can be better trained to learn complex images. The structure of an OD matrix can be viewed as an image where each matrix cell represents an image pixel. In summary, each CNN consists of three layers: convolutional layer, pooling layer, and fully connected layer. For more details, refer to previous studies on this topic [11,38,39,45]. To introduce data into CNN, the existing tabular data were converted to a 2×21 image. The first two rows show the characteristic of the origin and destination zone data. The proposed layers include the input layer that consists of two 2D convolutional layers, a pooling layer in the form of max pooling, and finally, the flatten layer, which is used to reduce the dimensions to one. The output of this step is linked to a fully connected structure that corresponds exactly to the MLP network explained in the previous section. For training and using the neural network model, the Keras library in the Python programming language was implemented [61].

## 5. Results

**Figure 8:**
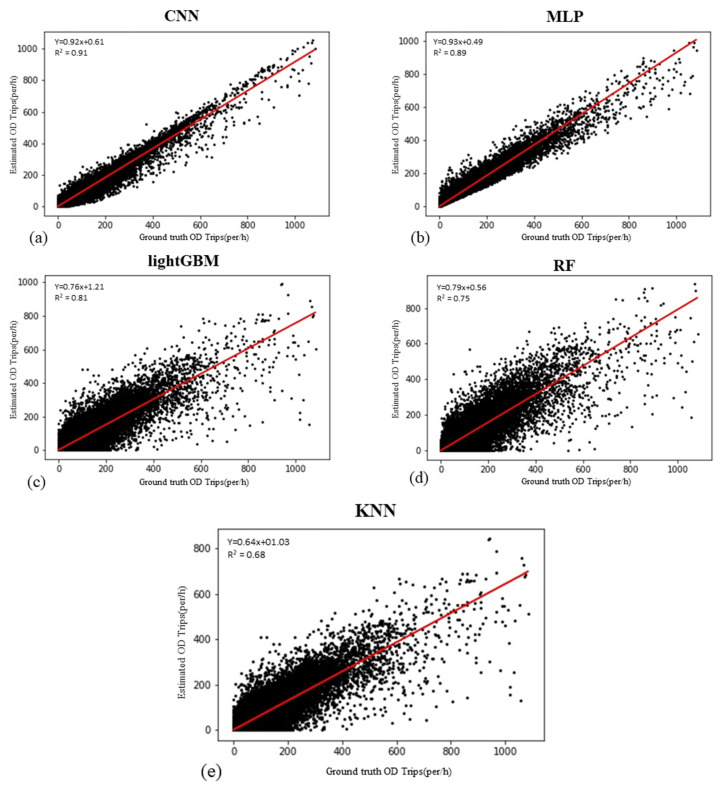
$R^{2}$ values of test data for developed models. (**a**) Estimated CNN model vs. Ground truth OD trips, (**b**) Estimated MLP model vs. Ground truth OD trips, (**c**) Estimated LightGBM model vs. Ground truth OD trips, (**d**) Estimated RF model vs. Ground truth OD trips, (**e**) Estimated KNN model vs. Ground truth OD trips.

The data set examined in this study consists of 11,726,424 hourly origin–destination records. Each record includes the number of daily trips between an origin and destination every hour. The model’s dependent variable is the OD matrix of CUTS, and the 21 variables listed in Table 2 are considered to be independent variables. In this regard, 75% of records were used for training, and the rest were used for testing the trained models. These percentages of dividing were considered for every model mentioned in the previous section. All input and output data were normalized to use in the models. The parameters of each model were tuned according to the best-fit model (maximum $R^{2}$ value). The models with different combinations of parameters were trained to obtain the optimal values of the parameters in each method. The trained models were used to predict the hourly OD matrix using the test data in the next step. Figure 8 shows the $R^{2}$ values for the test data of the developed models.

As shown in Figure 8, the CNN model with the value of $R^{2}=0.91$ fitted in the best way for the test data. The MLP model with the value of $R^{2}=0.89$ and the LightGBM model with $R^{2}=0.81$, are the next two best-fit models. In the following, statistical and structural indicators are examined to select the most accurate model for predicting the hourly OD demand matrix.

### 5.1. Comparison between Developed Models Based on Statistical Indicators

Statistical indicators, such as root mean square error (RMSE) and mean absolute percentage error (MAPE), were used to evaluate the performance of models. In addition, to evaluate the possibility of overfitting in the models, the $R^{2}$ values were examined separately for the training and test data. A comparison between the performance parameters of the developed models can be seen in Table 3.

As shown in Table 3, the KNN model exhibits the most unfavorable performance. As mentioned before, this model is one of the simplest ML models. The difference in $R^{2}$ values between the training data and test data show an overfitting problem in this model. The maximum $R^{2}$ value is observed for the test data of the CNN model. The model also has the lowest RMSE and MAPE values. The MAPE value of the CNN model is about 3% lower than the MAPE value for the MLP model and 5% lower than the MAPE value for the LightGBM model. Moreover, the CNN model has about 30% lower error than the basic ML models such as KNN. According to the RMSE indicator, the CNN model has about 10% better performance than the MLP model, while this advantage is about 16.5% compared with the LightGBM model. The best performance can be attributed to the CNN model. This model also shows the slightest difference in $R^{2}$ between the test and training data, indicating the least overfitting occurred in this model. The LightGBM model, which shows acceptable results in the training data, cannot predict the OD matrix reliably for the test data. Following the CNN model, the MLP model shows the best results. The structure of the CNN model justifies its superiority in estimating the OD demand matrix.

**Figure 9:**
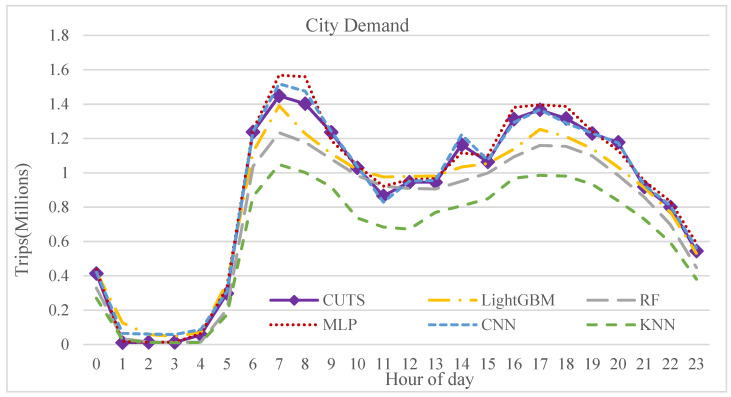
Comparison between developed models according to hourly distribution.

**Figure 10:**
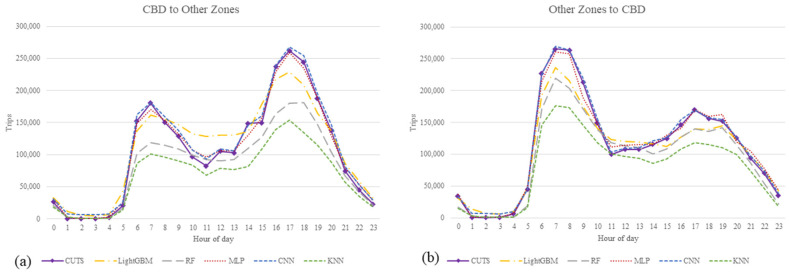
Comparison between developed models according to hourly distribution for CBD of Tehran. (**a**) temporal distribution of trips from CBD to other zones, (**b**) temporal distribution of trips from other zones to CBD.

Figure 9 and Figure 10 show the prediction results of the models in the form of temporal distribution. Figure 10 shows the trips predicted by the developed models from the CBD of Tehran as an origin to other zones as a destination and vice versa.

As shown in Figure 9, the travel distribution pattern between different zones at various times of a day is better matched in the CNN and MLP models than other developed models. These two models reasonably predict travel demand, particularly during peak hours, with proper accuracy. Furthermore, as shown in Figure 10, the best model of this study well recognizes the pattern of travels between specific zones, such as travels from/to the CBD of the city. The high demand for travels during the peak hours in the morning to the CBD of the city, and in reverse, the demand for leaving the CBD to other zones during the evening peak hours are well predicted by the CNN and MLP models.

### 5.2. Structural Comparison of Predicted Matrices

In addition to statistical indicators (such as RMSE and MAPE) for comparing the predicted and ground truth OD matrices, other methods have also been developed to understand the OD matrices’ structural similarity/dissimilarity. In these methods, in addition to numerical comparison of cells in two matrices (similar to statistical methods), the distribution of travels from different origins to different destinations or, in other words, the structure of the two matrices is also compared. One of these methods is the mean structural similarity index method (MSSIM) [13]. According to this method, the structural similarity between the two matrices varies from −1 to 1. A value of 1 denotes that the two matrices are exactly the same. When comparing the two matrices, the closer the structural similarity is to 1, the more the two matrices are similar from both numerical and structural perspectives. In this study, the OD matrices predicted by different models structurally compared with the ground truth OD matrix. The estimated hourly OD matrices were compared to the corresponding ground truth OD matrix in that hour to perform this comparison. The MSSIM values were then averaged for 24 h. The results of this comparison can be seen in Table 4.

According to Table 4, the CNN model has the highest structural similarity (MSSIM value equals 0.93) among the developed models. Thus, the matrix predicted by the CNN model has the highest similarity with the ground truth OD matrix from both numerical and structural perspectives. Interestingly, in comparison of MLP and LightGBM models, although the MLP model predicts the OD matrix better in terms of statistical and structural indicators, the LightGBM model exhibits a much closer result in terms of structural similarity. The unfavorable performance of the RF and KNN models is also visible in this comparison.

## 6. Conclusions

In this research, the convolutional neural network (CNN) is investigated as the most accurate model for estimating the origin–destination demand matrix in the Tehran metropolis. This study used the big data analysis concept based on the gathered data of ITS sources to estimate the hourly (dynamic) OD matrix of trips between TAZs. Data used in this study includes traffic flow in zones by ANPR cameras, entries to and exits from subway stations by SFCs, Loop detector traffic count in intersections by SCATS, the OD matrix from GPS data of Neshan navigation software, and characteristics of TAZs. The diversity and the large volume of usable data made ML methods useful to determine the most efficient model for predicting the hourly OD matrix of trips. In this regard, five different ML models (from primary to advanced models based on deep learning techniques) were trained on a target OD matrix in terms of person trip, adapted from CUTS in Tehran. The results of the development of the mentioned models are summarized as follows:

1. Among the developed models, the best results belong to the CNN and MLP models due to higher $R^{2}$ values for the test data, respectively. Overfitting is quite evident in rudimentary ML models such as KNN.
2. Comparing the models developed based on the statistical indicators, the CNN model has higher accuracy in estimating the OD matrix of trips for the test data according to the RMSE and MAPE values. The CNN model error is 10% less than the MLP model and 16.5% less than the LightGBM model. The superiority of the CNN model over the rudimentary ML models such as KNN was distinctly observed. The MLP model again ranked second in this comparison.
3. The structural comparison of two matrices reveals new aspects of similarity or difference between the matrices. In addition to the statistical indicators, this study compared the matrices structurally predicted by the ML models with the ground truth OD matrix. For this purpose, the MSSIM index was used to calculate the structural similarity between the two matrices. The highest structural similarity in the developed models was observed between the OD matrix of the CNN model and the ground truth OD matrix.

Accordingly, it could be concluded that neural networks have an undiscussable superiority in predicting the OD demand matrix among various ML techniques. Among the neural networks developed in previous studies, according to the structural nature of the convolutional neural network and the results of this study, these models make the most accurate predictions. This study shows that the CNN model provides the best results numerically (the value of each matrix cell) and structurally to estimate the OD matrix.

It should be noted that ML models act as a black box, and thus, no accurate interpretation of the relationship between input variables and output data can be extracted. Therefore, the interpretation of models under varying conditions is always prone to difficulties. As the process of data collection and processing becomes apparent to transportation experts, modelers may be able to use new techniques to analyze the data. Furthermore, completing additional information (e.g., license plate numbers of vehicles for the ANPR data or the profile of navigation software users) along with these data may allow for better analysis.

## Figures and Tables

**Table 1:** Characteristics of traffic analysis zones.

N-Variable	Feature
1	Population
2	Car ownership per capita
3	Employee population
4	Area of administrative land-use
5	Area of commercial land-use
6	Distance between zones

**Table 2:** Adjusted $R^{2}$ value for independent variables.

No.	Variables	$\mathrm{Adj}.\;R^{2}$	No.	Variables	$\mathrm{Adj}.\;R^{2}$
1	Population_O	0.213	12	Distances	0.41
2	Population_D	0.211	13	Neshan OD	0.54
3	Car ownership per capita_O	0.195	14	ANPR_Count_O	0.328
4	Car ownership per capita_D	0.192	15	ANPR_Count_D	0.347
5	Employee population_O	0.110	16	SCATS_Count_O	0.254
6	Employee population_D	0.121	17	SCATS_Count_D	0.208
7	Area of administrative land-use_O	0.094	18	SFC_En_O	0.389
8	Area of administrative land-use_D	0.124	19	SFC_En_D	0.359
9	Area of commercial land-use_O	0.155	20	SFC_Ex_O	0.346
10	Area of commercial land-use_D	0.157	21	SFC_Ex_D	0.339
11	Time of day	0.204			

**Table 3:** Performance evaluation of developed models.

Models	*R*^2^ Train	*R*^2^ Test	RMSE Train	RMSE Test	MAPE% Train	MAPE% Test
KNN	1	0.6832	0	8451.72	0	63.21
Random Forest	0.9648	0.7532	4321.65	7943.42	26.23	45.76
LightGBM	0.9721	0.8132	4065.93	6762.18	23.65	39.51
MLP	0.9521	0.8932	4654.61	6286.95	28.78	37.32
CNN	0.9681	0.9132	4156.73	5634.46	25.96	34.54

**Table 4:** Structural comparison of developed models by MSSIM method.

KNN	RF	LightGBM	MLP	CNN
0.64	0.73	0.86	0.87	0.93

## Data Availability

The datasets are available from the corresponding author on reasonable request.
